# Introgressive replacement of natives by invading *Arion* pest slugs

**DOI:** 10.1038/s41598-017-14619-y

**Published:** 2017-11-02

**Authors:** Miriam A. Zemanova, Eva Knop, Gerald Heckel

**Affiliations:** 1 Computational and Molecular Population Genetics, Institute of Ecology and Evolution, University of Bern, Baltzerstrasse 6, CH-3012 Bern, Switzerland; 2 Community Ecology Group, Institute of Ecology and Evolution, University of Bern, Baltzerstrasse 6, CH- 3012 Bern, Switzerland; 3 Swiss Institute of Bioinformatics, Genopode, CH-1015 Lausanne Switzerland

## Abstract

Hybridization with invasive species is one of the major threats to the phenotypic and genetic persistence of native organisms worldwide. *Arion vulgaris* (syn. *lusitanicus*) is a major agricultural pest slug that successfully invaded many European countries in recent decades, but its impact on closely related native species remains unclear. Here, we hypothesized that the regional decline of native *A. rufus* is connected with the spread of invasive *A. vulgaris*, and tested whether this can be linked to hybridization between the two species by analyzing 625 *Arion* sp. along altitudinal transects in three regions in Switzerland. In each region, we observed clear evidence of different degrees of genetic admixture, suggesting recurrent hybridization beyond the first generation. We found spatial differences in admixture patterns that might reflect distinct invasion histories among the regions. Our analyses provide a landscape level perspective for the genetic interactions between invasive and native animals during the invasion. We predict that without specific management action, *A. vulgaris* will further expand its range, which might lead to local extinction of *A. rufus* and other native slugs in the near future. Similar processes are likely occurring in other regions currently invaded by *A. vulgaris*.

## Introduction

The displacement of native species by invasive ones is a serious threat to biodiversity worldwide, and is becoming more frequent due to the increasing number of species introductions^1,2^. Among the mechanisms of such displacement are very often competition or predation^3,4^. However, invasive species can also displace native organisms genetically, through hybridization and introgression^5,6^. This mixing of gene pools and potential loss of genotypically distinct species is especially problematic for rare organisms coming into contact with more abundant ones^7,8^.

Hybridization might have different outcomes for the native and invasive species. Even though hybridization can in some cases contribute to generating species diversity^9^, the hybrid offspring could also be infertile or have reduced fitness, which may lead to decline and even extinction of the native species’ populations^10,11^. Moreover, hybridization can result in speciation reversal, and change the functional roles of organisms in the ecosystem^12,13^. For the invasive species, hybridization may provide advantages by increasing their invasiveness or adaptive potential to local environments^14–16^. The consequences of hybridization can be particularly challenging for conservation biology, because it is difficult to develop management strategies for hybrids of endangered species^17^. Therefore, to be able to protect native organisms and prevent spread of invaders, it is crucial to understand whether hybridization has occurred as well as what are its likely impacts.

Most studies on the impacts of hybridization between invasive and native species have been conducted in plants (e.g. ref.^4,5^), with only a few in animals (e.g. ref.^15,18^). Among the major animal taxonomic groups, molluscs have the highest number of documented extinctions, which has been attributed in part to interactions with other mollusc species invading native species ranges^19^. However, the role of hybridization with invasive species in molluscs remains poorly understood.

The hermaphroditic slug *Arion vulgaris* Moquin-Tandon 1855 (also referred to as non-topotype *A. lusitanicus* Mabille 1868) is considered one of the 100 most invasive species in Europe^20^, being a serious pest both in agriculture and gardening^21,22^. It has spread and become established in many European countries since the 1950’s^23–25^, but see ref.^26^ and represents an excellent case for studying the genetic impacts on native species, such as *A. rufus* (Linnaeus 1758). This closely related native slug used to be abundant in forests and meadows^27^, but recently has been listed as “vulnerable” on the Red List of endangered species in several European countries^28,29^. It has been reported that when *A. vulgaris* enters an area, the populations of *A. rufus* begin to decline, and hybridization is suspected to be one of the underlying mechanisms^30,31^.

The possibility and extent of hybridization among the three large *Arion* species *– A. vulgaris*, *A. rufus*, and *A. ater* (Linnaeus 1758) – has been sparked by and questioned following the observation of intermediate morphological phenotypes in the field and mating experiments under experimental laboratory conditions^32,33^. Initial genetic investigations indicated hybridization between the invasive *A. vulgaris* and native *A. ater* or *A. rufus* in particular^34–36^, but low-resolution genetic markers and sample sizes limited the conclusiveness. Thus, it remains unanswered how frequently hybridization among the invasive *A. vulgaris* and the native *A. rufus* occurs, and whether hybrid offspring can persist and have a lasting impact on natural populations.

**Figure 1:**
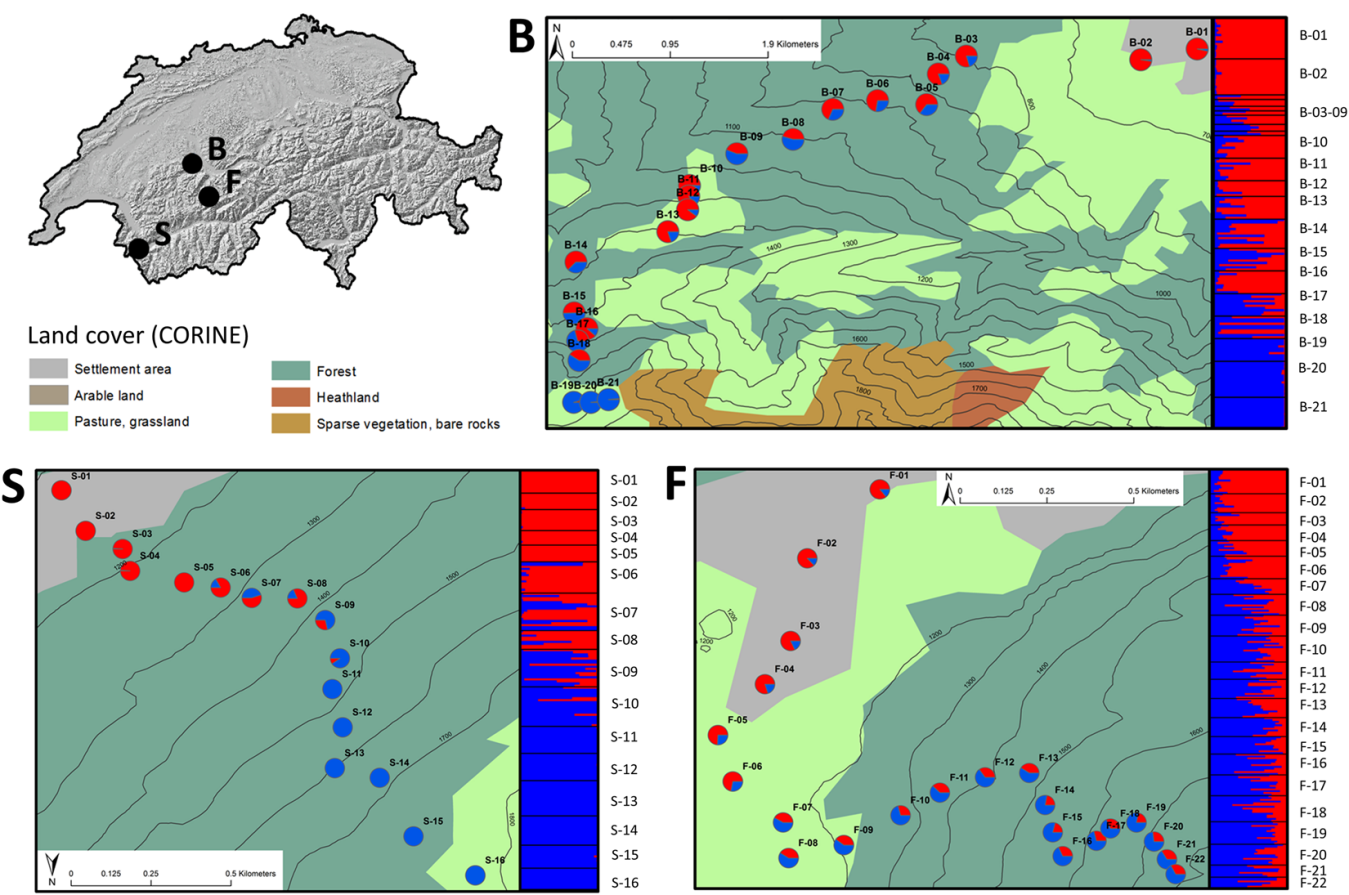
Genetic admixture between *Arion vulgaris* and *A. rufus* along the altitudinal transects Blumenstein (**B**), Salvan (**S**) and Filfalle (**F**) in Switzerland. Pie charts represent average assignment probabilities from STRUCTURE for the two species based on nuclear DNA. The genetic cluster representing *A. vulgaris* is shown in red, *A. rufus* in blue. Individual membership coefficients are displayed in the bar plots: each individual is represented by a single horizontal bar, with sampling location labels shown on the right. Sampling locations are separated by a black line. The patterns suggest the presence of a zone of genetic admixture at intermediate altitudes for S and B. The Filfalle transect shows signs of more extensive admixture between the invasive and the native *Arion* species. This figure was produced using the ArcGIS software version 10.2.2 (www.esri.com/software/Arcgis).

In order to characterize the interactions between invasive *A. vulgaris* and native *A. rufus*, we examined the zone of contact of these two species in three different locations. In Switzerland, *A. vulgaris* has established a continuous distribution below 1000 m above sea level (a.s.l.) according to morphological assessment^28^ and given its synanthropic nature this species is often present in cities and villages^28,37^. However, species identification based on external morphology may be misleading^36^ and genetic analyses of the slug invasion are lacking in Switzerland and elsewhere. Nonetheless, the most abundant large *Arion* slugs in cultivated areas in the Swiss lowlands are most likely *A. vulgaris*, while the native *A. rufus* is nowadays thought to be limited to higher altitudes^28^. In order to determine if and where in the landscape the two species are in contact, we sampled slugs along altitudinal transects ranging from urbanized areas in valleys to relatively pristine habitats on mountain tops (Fig. 1). Given that the first occurrence record of *A. vulgaris* in Switzerland dates back to 1955^38^, the potential interactions between the invading and native species may have occurred over about 60 generations in the region, and our investigation could thus provide valuable insights into the genetic impacts of a recent invasion process. Specifically, in this study we assessed i) whether there is evidence of past or recent hybridization between the two species, and ii) whether there is consistency between genetic and morphological species assignment, with the potential implications for management of the invasive and conservation of the native species.

## Results

### Species identification based on mtDNA and morphology

**Figure 2:**
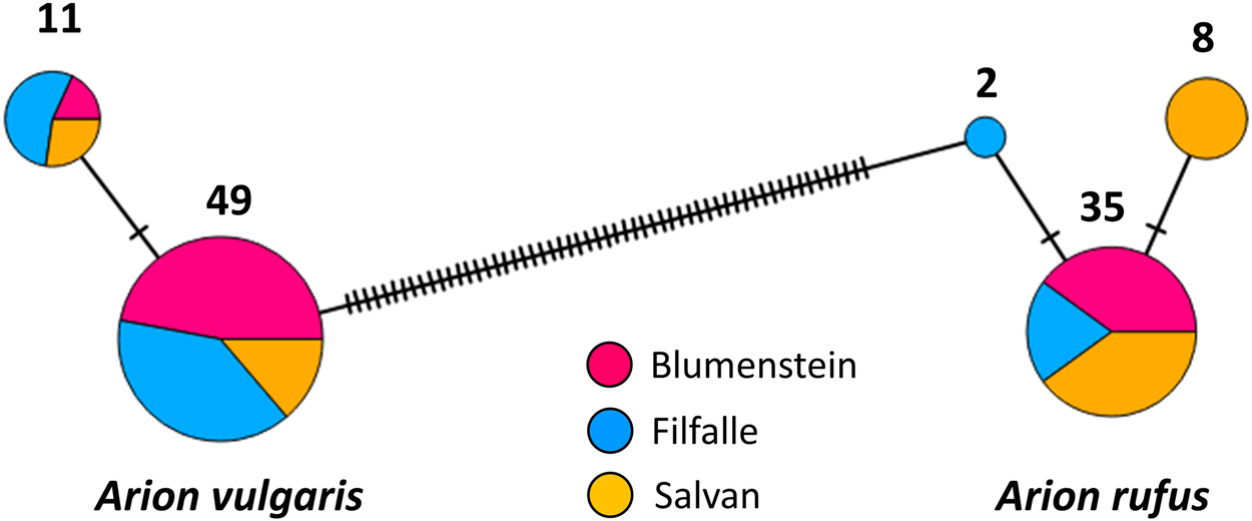
Statistical parsimony haplotype network of the 105 ND1 sequences of *A. vulgaris* (left) and *A. rufus* (right) sampled in the Blumenstein, Filfalle and Salvan transects. Each circle represents one unique haplotype, and its size is proportional to the number of individuals with the particular haplotype. Number of individuals with the same haplotype is indicated above the circles. Mutations are shown as hatch marks. Number of individuals and sampling locations are listed in Tables 1–3.

Phylogenetic reconstructions clearly distinguished two clades representing the invasive and native species (Fig. S3). Forty-five individuals carried *A. rufus* and 60 *A. vulgaris* mtDNA haplotypes, with extensive sharing of haplotypes between the three altitudinal transects. Our sequencing yielded two different haplotypes for *A. vulgaris* and three haplotypes for *A. rufus*, and these differed in at least 60 nucleotides between the species (Fig. 2). Mitochondrial DNA from both species was detected in pasture and forested habitats but there was strong variation between transects and altitude (Tables 1, 2 and 3).

**Table 1:** Sampling locations in the Blumenstein altitudinal transect with the number of samples analyzed for nuclear DNA (nucDNA) and identified with mitochondrial ND1 marker: *A. vulgaris* (ND1: AV) or *A. rufus* (ND1: AR).

Location	Latitude	Longitude	Elevation	nucDNA	ND1: AV	ND1: AR	M: AV	M: AR	M: H	M: np
B-01	46.742	7.522	664	18	2	—	2	—	—	—
B-02	46.741	7.517	669	16	2	—	2	—	—	—
B-03	46.742	7.502	782	2	1	—	1	—	—	—
B-04	46.740	7.499	829	3	2	1	2	—	—	1
B-05	46.737	7.498	915	2	—	—	—	—	—	—
B-06	46.738	7.494	941	2	—	—	—	—	—	—
B-07	46.737	7.490	1013	4	2	—	2	—	—	—
B-08	46.734	7.487	1073	3	—	—	—	—	—	—
B-09	46.733	7.482	1131	2	—	—	—	—	—	—
B-10	46.730	7.478	1211	10	2	—	2	—	—	—
B-11	46.729	7.477	1244	10	3	—	2	—	1	—
B-12	46.728	7.477	1282	7	2	—	2	—	—	—
B-13	46.726	7.476	1361	10	3	—	3	—	—	—
B-14	46.724	7.468	1385	13	1	1	1	1	—	—
B-15	46.719	7.467	1466	10	—	2	—	2	—	—
B-16	46.718	7.469	1522	10	2	—	2	—	—	—
B-17	46.717	7.468	1557	10	1	—	1	—	—	—
B-18	46.715	7.468	1615	10	1	1	1	—	—	1
B-19	46.711	7.467	1760	10	—	3	—	2	—	1
B-20	46.711	7.469	1822	16	—	3	—	2	—	1
B-21	46.712	7.470	1872	15	—	3	—	1	—	2

Morphological assessment is also listed (M: AV – *A. vulgaris*, M: AR – *A. rufus*, M: H – putative hybrid, M: np – morphological assessment not possible).

**Table 2:** Sampling locations in the Salvan altitudinal transect with the number of samples analyzed for nuclear DNA (nucDNA) and identified with mitochondrial ND1 marker: *A. vulgaris* (ND1: AV) or *A. rufus* (ND1: AR).

Location	Latitude	Longitude	Elevation	nucDNA	ND1: AV	ND1: AR	M: AV	M: AR	M: H	M: np
S-01	46.111	7.007	1123	11	3	—	3	—	—	—
S-02	46.112	7.006	1187	8	—	—	—	—	—	—
S-03	46.113	7.005	1244	10	2	—	2	—	—	—
S-04	46.113	7.005	1273	8	—	—	—	—	—	—
S-05	46.114	7.003	1291	8	—	—	—	—	—	—
S-06	46.114	7.002	1315	15	2	2	1	1	—	2
S-07	46.114	7.001	1357	18	—	6	1	3	1	1
S-08	46.114	7.000	1407	8	2	—	2	—	—	-
S-09	46.115	6.999	1449	18	1	1	1	—	—	1
S-10	46.116	6.999	1515	19	—	2	1	1	—	—
S-11	46.117	6.999	1559	13	—	—	—	—	—	—
S-12	46.118	6.999	1623	13	—	2	—	1	—	1
S-13	46.119	6.999	1674	17	—	—	—	—	—	—
S-14	46.119	6.998	1720	14	—	—	—	—	—	—
S-15	46.121	6.997	1767	11	—	4	—	3	1	—
S-16	46.122	6.995	1845	10	—	5	—	4	1	—

Morphological assessment is also listed (M: AV – *A. vulgaris*, M: AR – *A. rufus*, M: H – putative hybrid, M: np – morphological assessment not possible).

**Table 3:** Sampling locations in the Filfalle altitudinal transect with the number of samples analyzed for nuclear DNA (nucDNA) and identified with mitochondrial ND1 marker: *A. vulgaris* (ND1: AV) or *A. rufus* (ND1: AR).

Location	Latitude	Longitude	Elevation	nucDNA	ND1: AV	ND1: AR	M: AV	M: AR	M: H	M: np
F-01	46.494	7.673	1173	14	3	—	3	—	—	—
F-02	46.492	7.671	1174	11	—	—	—	—	—	—
F-03	46.490	7.671	1175	7	3	—	3	—	—	—
F-04	46.489	7.670	1181	9	—	—	—	—	—	—
F-05	46.488	7.669	1182	9	3	—	3	—	—	—
F-06	46.486	7.670	1183	13	—	—	—	—	—	—
F-07	46.485	7.671	1191	9	2	1	2	—	—	1
F-08	46.484	7.671	1223	12	—	—	—	—	—	—
F-09	46.485	7.672	1289	12	1	2	2	—	—	1
F-10	46.485	7.674	1350	15	—	—	—	—	—	—
F-11	46.486	7.675	1390	10	1	2	1	—	1	1
F-12	46.486	7.676	1434	11	—	—	—	—	—	—
F-13	46.487	7.677	1483	11	3	—	2	—	—	1
F-14	46.486	7.678	1546	11	—	—	—	—	—	—
F-15	46.485	7.678	1585	10	3	—	3	—	—	—
F-16	46.484	7.678	1630	12	—	—	—	—	—	—
F-17	46.485	7.679	1664	12	2	—	1	—	—	1
F-18	46.485	7.679	1685	15	—	—	—	—	—	—
F-19	46.485	7.680	1708	13	2	—	2	—	—	—
F-20	46.485	7.680	1732	12	1	1	1	—	1	—
F-21	46.484	7.681	1760	7	—	—	—	—	—	—
F-22	46.484	7.681	1801	6	2	3	2	2	1	—

Morphological assessment is also listed (M: AV – *A. vulgaris*, M: AR – *A. rufus*, M: H – putative hybrid, M: np – morphological assessment not possible).

Based on internal morphology we were able to classify 89 out of the 105 individuals sequenced for mtDNA. The remaining 16 specimens had immature genitalia or their preservation state did not permit a clear identification. Genital morphology allowed us to identify 59 individuals as *A. vulgaris*, 23 as *A. rufus*, whereas the remaining seven showed intermediate morphological characteristics (Tables 1, 2, 3 and 4).

**Table 4:** Comparison of species assignment with nuclear DNA (nucDNA), mitochondrial DNA (mtDNA) and morphology across all three transects (see Figure S4 for further information).

nucDNA	mtDNA	Morphology	Nr.
*A. vulgaris*	*A. vulgaris*	*A. vulgaris*	20
*A. vulgaris*	*A. vulgaris*	hybrid	—
*A. vulgaris*	*A. vulgaris*	*A. rufus*	—
*A. vulgaris*	*A. rufus*	*A. vulgaris*	2
*A. vulgaris*	*A. rufus*	hybrid	—
*A. vulgaris*	*A. rufus*	*A. rufus*	—
hybrid	*A. vulgaris*	*A. vulgaris*	30
hybrid	*A. vulgaris*	hybrid	2
hybrid	*A. vulgaris*	*A. rufus*	2
hybrid	*A. rufus*	*A. vulgaris*	4
hybrid	*A. rufus*	hybrid	2
hybrid	*A. rufus*	*A. rufus*	2
*A. rufus*	*A. vulgaris*	*A. vulgaris*	3
*A. rufus*	*A. vulgaris*	hybrid	—
*A. rufus*	*A. vulgaris*	*A. rufus*	—
*A. rufus*	*A. rufus*	*A. vulgaris*	—
*A. rufus*	*A. rufus*	hybrid	3
*A. rufus*	*A. rufus*	*A. rufus*	19

The number of slugs in each category is given.

### Signs of admixture and frequency of hybrids

All microsatellite loci were highly polymorphic with an average of 15 alleles per locus (ranging from 9 to 26, Table S1). Using the threshold values obtained in HYBRIDLAB, we detected the presence of both non-admixed and admixed individuals in each transect with large differences regarding the frequency and distribution of hybrids. Out of the 183 individuals analysed in the Blumenstein transect, 49 were identified as non-admixed *A. vulgaris*, 54 as non-admixed *A. rufus* and 80 as hybrids with various levels of admixture. In the Salvan transect, 66 individuals were identified as pure *A. vulgaris*, 99 as pure *A. rufus* and 36 as hybrids. In the Filfalle transect, there were 25 and 31 pure *A. vulgaris* and pure *A. rufus*, respectively, and 185 individuals identified as hybrids. Overall, we detected five cases (1–2 per transect) of mitonuclear discordance in both directions, i.e. species assignment for these individuals based on the autosomal loci was different from mtDNA (Table 4, Fig. S4). Genetic differentiation between non-admixed *A. vulgaris* and *A. rufus* in the three study regions was significant (p < 0.001) and relatively high in Blumenstein (F_ST_ = 0.425) and Salvan (F_ST_ = 0.475) but lower in Filfalle (F_ST_ = 0.186).

### Spatial patterns in nuclear DNA

**Figure 3:**
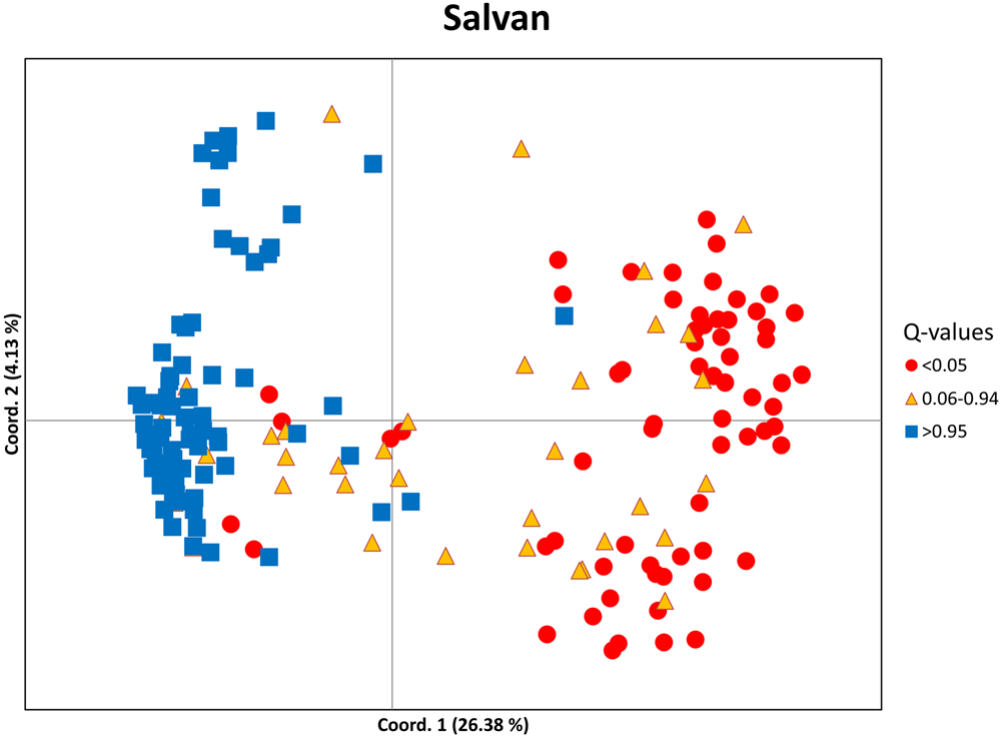
Results of the principal coordinate analysis for *Arion* sp. in the Salvan transect based on microsatellite genotypes. Individuals are colour-coded based on the HYBRIDLAB results: red – pure *A. vulgaris* (q-value < 0.05), blue – pure *A. rufus* (q-value > 0.95), yellow – admixed individuals (q-value 0.06–0.94). The percentage of the total variation in the dataset explained by each principal coordinate is given in parentheses. See Fig. S3 for the Blumenstein and Filfalle transects.

The PCoA separated most non-admixed *A. vulgaris* in the Salvan transect clearly from non-admixed *A. rufus* (defined by the thresholds identified in HYBRIDLAB) with likely hybrids in between (Fig. 3). The first and second axis accounted for 26% and 4% of the variation, respectively. The separation between the species was less pronounced in the Blumenstein transect and practically non-existent in the Filfalle transect (Fig. S5).

Combining the three transect datasets for the Bayesian analysis, there was extensive structure among and within transects with gradual transitions between genotype clusters in each study region (Fig. S6). The Evanno approach suggested that the most likely number of genetic clusters was K = 2 for Blumenstein and Salvan, reflecting the results of mtDNA analysis regarding the general distribution of the two species and the PCoA. The genetic cluster representing *A. vulgaris* was present in the lowest parts of the sampling transects and *A. rufus* in the highest parts with different levels of admixture at approximately intermediate altitudes (Fig. 1). For the Filfalle transect, the Evanno approach suggested K = 8 as the most likely number of clusters. However, given the presence of mtDNA from two species only, we used K = 2 for further analyses of the Filfalle dataset. This showed that a majority of *A. vulgaris* genotypes were found at low altitudes. Despite different levels of admixture in the three study regions, all results were consistent among independent STRUCTURE runs.

**Figure 4:**
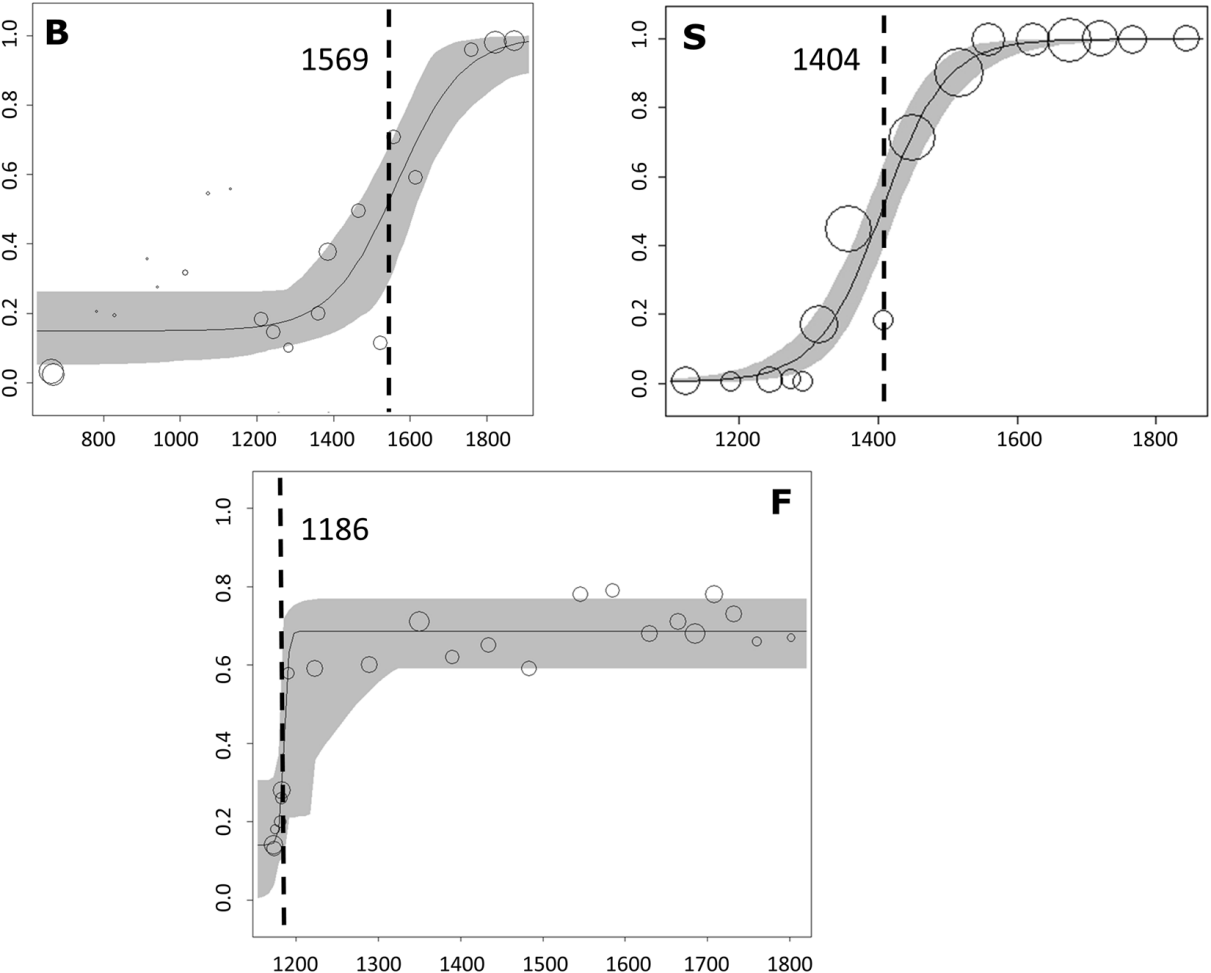
Results of the cline analyses for the three altitudinal transects (**B** – Blumenstein, **S** – Salvan, **F** – Filfalle) based on the average q-value for each sampling location obtained with STRUCTURE. Elevation in metres above sea level is on the x-axis and the probability of belonging to the *A. rufus* cluster (q-value) on the y-axis. Circles correspond to sampling locations and their size is proportional to the number of samples within a cline. The 95% credible cline region is indicated in grey. Filfalle shows signs of more extensive admixture between the invasive and the native *Arion* species also at the highest altitudes. The altitude of the centre of the cline is indicated by a dashed line.

When we estimated the position of the contact between species along the altitudinal transects with *hzar*, all fitted clines converged to the same values showing a single likelihood peak each. We observed relatively steep genetic clines of species transition with significant differences among the transects (Fig. 4). The cline centres were estimated to be located at 1,569 m a.s.l. in Blumenstein, 1,404 m a.s.l. in Salvan and at 1,186 m a.s.l. in Filfalle (Table S2). In addition to having the cline centre at the lowest altitude, Filfalle also displayed a very low estimated cline width (13 m of elevational difference) in comparison to Blumenstein (320 m) and Salvan (185 m; Table S2).

## Discussion

In this study, we provided a landscape level perspective on the spatial genetic consequences of the early stages of contact between an invasive and native animal species. Genetically confirmed *A. vulgaris* was predominantly found in disturbed areas around settlements and *A. rufus* in more natural habitats at higher altitude which is consistent with a human contribution e.g. through inadvertent introduction at the origin of the invasion process. Considering the overlapping external morphology between the two species, we propose molecular techniques be used as the primary method of species identification for these taxa.

Our analyses provided the first large-scale evidence of hybridization between the invasive *A. vulgaris* and native *A. rufus* under natural conditions. This supports previous suggestions that hybridization is possible (e.g. ref.^32,34^), despite the divergence time among extant *Arion* spp. slugs estimated at approximately 5 Mya^39^. More importantly, this study shows that hybridization can be common where the two species co-occur, and through our design of small-scale landscape level transects in comparison with other studies spanning tens or hundreds of km (e.g. ref.^40,41^) we were able to precisely determine the position of the *A. vulgaris* invasion front.

The detection of individuals with different levels of admixture in all three regions suggests that interspecific mating is not limited to the first generation of hybrids. This is corroborated by the observation of a few individuals displaying mitonuclear discordance in both directions which requires several successful reproduction events of hybrids and backcrosses over multiple generations (Table 4; Fig. S4; ref.^42,43^).

Evidence of hybridization was observed in all three study regions but genetic admixture seems to be particularly extensive in the Filfalle transect. This could be explained by at least two scenarios. First, the two species have been in contact for more time than in the other transects. It is unknown when exactly the two species came into contact in the respective regions but the shared mtDNA haplotypes between regions (Fig. 2) – that are also frequent in the invaded ranges elsewhere in Europe^25^ – provide no indication of a substantially different local invasion history in Filfalle. Second, it is possible that the *A. vulgaris* slugs that invaded this region were already admixed which would explain the overall lower level of differentiation in nuclear DNA between the taxa in this region. A third possibility would be two consecutive invasions of the region. The first invasion could have led to extensive admixture even at high altitudes while the second one with mostly pure *A. vulgaris* is still ongoing. Very specific demographic analyses might be able to differentiate between these invasion scenarios but this would require vast genomic data sets^44^.

Theoretical work by Currat *et al*.^7^ predicts that in the zone of contact, invaders could have experienced more introgression at selectively-neutral genes than the native species. These predictions have been supported in several empirical studies (e.g. ref.^8,45,46^), which however investigated hybrid zones that have existed for a long time. In our system, the invasion of *A. vulgaris* in Switzerland started approximately 60 years ago^38^, and we did not detect a pattern of asymmetrical introgression. It is unclear if this will build up over time and potentially lead to larger geographic regions with introgressed individuals (e.g. ref.^42,47^). Accordingly, very little pronounced patterns are expected in this regards for the regions of Europe that were more recently invaded or are currently being invaded by *A. vulgaris* ^25^.

Because our study represents to our knowledge the first genetic assessment of the levels of introgression in *Arion* sp. slugs at the landscape scale, we do not have a baseline from an earlier time point to which these levels could be compared. We thus cannot determine with certainty whether the trend of introgression is declining or increasing. However, several studies have suggested that the propensity to hybridize might be influenced by relative abundance^48,49^, and if the more abundant *A. vulgaris* continues to spread, we predict that it will eventually lead to introgressive replacement of *A. rufus*. Such genetic replacement has been described for example in native newts^8^, crayfish^50^ and mussels^51^.

Nevertheless, additional factors could be contributing to the disappearance of *A. rufus*. Invasive species are often considered competitively dominant: they might use food resources more rapidly and/or efficiently than native species^52^, exhibit rapid adaptation and spread^53^, faster growth rates^54^ and higher reproductive output^55^. Indeed, *A. vulgaris* seems to be able to use food resources more efficiently than native species^56,57^, and shows a more pronounced exploratory behaviour in novel environments^58,59^. Further, *A. vulgaris* seems to be able to cope with land use change and agricultural intensification better than the native species^60^. Together with hybridization, these factors might cause local extinction of *A. rufus* in the near future.

The two species mostly occurred in different habitats, as described by Rüetschi *et al*.^28^ based on morphological assessments. The contact between *A. rufus* and *A. vulgaris* is currently at intermediate altitudes (Fig. 1) with the cline centres at different elevations in each region (Fig. 4, Table S2), which might indicate differences in local invasion histories of *A. vulgaris* ^61,62^ as mentioned above. While the analogous highest altitude of slug occurrence in all study regions suggests environmental factors (e.g. temperature) constraining further spread of both species^57^, it would be interesting to implement long-term genetic monitoring to document any further expansion of *A. vulgaris* over time^62^.

Most of the individuals assigned by nucDNA as hybrids were morphologically identified as either species (Table 4). Species identification of *Arion* taxa is notoriously difficult especially regarding differentiation based on external morphology^36^, and hybridization adds another complication. Although morphological species assignment is still extensively used for *Arion* spp. slugs (e.g. ref.^63,64^), our results stress that identification should be primarily based on molecular techniques, particularly in regions where multiple species co-occur. In order to preserve the native, often endangered species, we encourage the use of non-invasive DNA sampling methods (e.g. ref.^65,66^) whenever possible.

Some of the observed discrepancy between genetic and internal morphological species assignment (Table 4) might be caused by phenotypic overlap, i.e. hybrids after the F1 generation could be difficult to tell apart from parental species^17,67^. In this case, the removal of morphologically indistinguishable hybrids from mixed populations would be impossible, and management efforts need to focus primarily on prevention of further spread in the landscape and new introductions of the invasive slug.

We have unequivocally established that *A. vulgaris* – one of the most destructive agricultural pest slugs in Europe – is able to hybridize with *A. rufus* and produce fertile offspring in the natural environment. Given the current rapid spread of *A. vulgaris* through vast areas of Europe, similar processes might be acting in other countries and closely related species although the time in contact is shorter in most regions and there is no evidence of “de-speciation” at larger scales yet^25^. Nevertheless, the invasion of the slug *A. vulgaris* may constitute a larger threat to native *Arion* species than previously considered.

## Materials and Methods

### Field sampling and morphological species identification

We chose three study regions (Blumenstein, Salvan and Filfalle) in the Swiss Prealps based on the occurrence records of *A. rufus* in the last 10 years^38^ (Fig. 1). In each study region, sampling along an elevational transect started in a settlement area and continued through mostly forested locations and pastures to higher altitudes (Fig. 1; Tables 1, 2 and 3). As the home range size of *A. vulgaris* has been estimated to be approximately 45 m^2^ ^68^, sampling locations within a transect were spaced at least 50 meters apart to increase the chance of capturing unrelated individuals. Along the transect Blumenstein, 183 individuals were sampled from 21 locations in the altitudinal range of 664–1,872 m a.s.l. (Table 1). The transect Salvan comprised 16 locations, yielding 201 individuals from altitudes between 1,123 and 1,845 m a.s.l. (Table 2), and in the transect Filfalle, we collected 241 individuals from 22 locations (altitude 1,173–1,801 m a.s.l.; Table 3). The highest altitudes in the transects constituted the upper limit of where we detected *Arion* slugs in the respective locations. In total, 625 slugs were analysed.

Slugs were collected directly in the field during rainy weather or at night, or by placing a trap designed by the authors (described in detail in Fig. S1) overnight. Slugs were killed by freezing at -20 °C for at least eight hours and the whole specimens were preserved in absolute ethanol. Slugs that were analysed with both mitochondrial and nuclear markers (105 individuals) were dissected and classified as either *A. vulgaris*, *A. rufus* or an intermediate form based on internal morphological traits of reproductive organs^37,69^. DNA was extracted from a small piece of foot tissue using proteinase K and the high-salt extraction method^70^. After dilution in double-distilled water, the DNA was stored at −20 °C.

### Mitochondrial DNA analyses

In order to confirm that both *A. vulgaris* and *A. rufus* were present in each region, we sequenced the mtDNA gene ND1 in 32–38 *Arion* sp. individuals per transect (Tables 1, 2 and 3), totalling 105 slugs. Locus specific primers MOL-NAD1F (5′-CGRAARGGMCCTAACAARGTTGG-3′) and MOL-NAD1R (5′-GGRGCACGATTWGTCTCNGCTA-3′) developed by Quinteiro *et al*.^39^ were used to amplify a 350 bp long fragment, following the protocol described in Zemanova *et al*.^25^. The sequencing reactions were visualized on an ABI Prism 3130 (Applied Biosystems).

To assign slug mtDNA to the native or invasive species, we aligned our novel ND1 sequences with reference sequences from our previous study^25^ covering the entire distribution range of the species: *A. vulgaris* sequences with the accession numbers KX834566, KX834594, KX834637 and *A. rufus* sequences with the accession numbers KX834609, KX834617, KX834665. We also used one *A. subfuscus* Draparnaud 1805 sequence (accession number AY316248) as outgroup for the phylogenetic analysis. DNA sequences were aligned in BIOEDIT 7.1.3^71^ and the number of haplotypes was determined in DNASP 5^72^. The phylogenetic tree was produced following the methodology described in Zemanova *et al*.^25^. In order to visualize relationships between haplotypes in either of the two species, we reconstructed a statistical parsimony haplotype network in POPART 1^73^.

### Nuclear DNA analyses

#### Genotyping with microsatellite markers

We genotyped all 625 *Arion* sp. individuals with fifteen nuclear microsatellite markers (nucDNA) ALU_02_3, ALU_06_4, ALU_11_2, ALU_12_2, ALU_13_2, ALU_30_2, ALU_34_2, ALU_37_2, ALU_60_2, ALU_76_2, ALU_79_2, ALU_86_2, ALU_88_2, ALU_92_2 and ALU_96_2^74^ in three primer mixes. The 10 µl PCRs contained approximately 100 ng DNA, 1 µl of primer mix, 5 µl of Qiagen multiplex kit and 3 µl of H_2_O. The PCR temperature profile was as follows: 15 min initial denaturation at 96 °C, followed by 32 cycles of denaturation at 94 °C for 30 sec, annealing at 57 °C for 1 min 30 sec, extension at 72 °C for 1 min 30 sec, and the final extension step of 30 min at 60 °C. The PCR product was diluted with 20 μl of distilled H_2_O and 1.2 μl of the diluted product were mixed with 12 μl of the internal size standard (GeneScan 500 LIZ, Applied Biosystems) to determine the size of alleles. The amplified fragments were separated on an ABI Prism 3130 Genetic Analyzer and fragment lengths were scored in GENEMAPPER 3.7 (Applied Biosystems). Approximately 10% of the samples were re-amplified and genotyped independently to ensure genotyping consistency.

#### Genetic diversity and estimates of admixture

The number of alleles per locus was calculated in GENALEX 6.5^75^. We also conducted a principal coordinate analysis (PCoA) in the same software to summarize major patterns of variation in the microsatellite genotype dataset. FSTAT 2.9.3.2^76^ was used to measure the average pair-wise level of genetic differentiation (F_ST_) between 50 individuals in each transect classified by the Bayesian analysis (see below) as either non-admixed *A. vulgaris* or *A. rufus*. We used Bayesian assignment implemented in STRUCTURE 2.3.4^77^ to identify genetic clusters in the *Arion* sp. samples. Analyses were run using the admixture model with correlated allele frequencies and without sampling location priors, with a burn-in period of 100,000 iterations followed by 1,000,000 MCMC iterations. The number of clusters (K) was set to range from 1 to 10, with 10 replicates for each K value. The optimal number of clusters was estimated using the Evanno approach^78^ implemented in Structure Harvester 0.6.94^79^. These analyses were run separately for each transect, as well as for all three transects together.

To identify the optimal threshold q-value (i.e. the probability of belonging to a genetic cluster) for distinguishing between non-admixed individuals and potential hybrids between the two species, we used Hybridlab 1.0^80^ to simulate parental and hybrid genotypes in each transect. The parental genotypes used for the simulations consisted of 50 individuals assigned to their genetic cluster with a q-value above 0.8. We simulated 10 data sets each with 100 generated genotypes of each parental and hybrid class (F1, F2, backcross with *A. vulgaris*, backcross with *A. rufus*). The simulated data sets were evaluated in STRUCTURE with K = 2 and 50,000 MCMC iterations following a burn-in period of 10,000. The accuracy and efficiency of assignment of the generated genotypes was calculated using the thresholds of 0.01, 0.05, 0.1, 0.2 and 0.3, following the procedure described in Vähä & Primmer^81^. The HYBRIDLAB results suggested that the best thresholds for delimiting non-admixed and hybrid individuals were 0.05 for Blumenstein and Salvan, and 0.15 for Filfalle (Fig. S2).

#### Cline analyses

To estimate the location and the spatial extent of admixture along the altitudinal transects, we conducted cline analyses with averaged q-values per sampling location using the statistical package *hzar* ^82^ implemented in R 3.1.1^83^. We modeled the cline shape using three equations^84–86^ that describe a sigmoid shape at the centre of the transition with two exponential decay curves on either side of the transition. We estimated the centre and width of each cline, and fitted three sets of three cline models using the Metropolis-Hasting algorithm. Model one had no scaling, model two had fixed scaling, and model three allowed the P_min_ and P_max_ (i.e. the bottom and top of a cline) to vary. We compared these models to a null model of no clinal transition. We ran each model for 100,000 iterations and assessed convergence and stability by visualizing the MCMC traces. We then selected the best-fit model based on the comparison of corrected AIC values.

### Data accessibility

DNA sequences are available in GENBANK under accession numbers MG253687-MG253791. Genotype data are available in Dryad under doi:10.5061/dryad.rb187.

## Electronic supplementary material

Supplementary information
