# Supplementary material for: Introgressive replacement of natives by invading Arion pest slugs
Source: Sci Rep. 2017 Nov 2;7:14908. doi: 10.1038/s41598-017-14619-y (PMC5668256; doi:10.1038/s41598-017-14619-y)
Supplement: Supplementary file 1 — Supplementary information [file 41598_2017_14619_MOESM1_ESM.pdf]

Supporting information for:

Introgressive replacement of natives by invading *Arion* pest slugs

Zemanova, Miriam A., Knop, Eva, Heckel, Gerald

Supplementary Tables

Table S1: Number of alleles per locus and sampling transect, and the total number of alleles across all transects.

Locus	Location			
	Blumenstein	Salvan	Filfalle	Total
ALU_12	13	10	15	21
ALU_34	12	6	14	20
ALU_37	15	8	17	18
ALU_60	6	7	7	9
ALU_06	13	13	11	20
ALU_76	5	9	9	12
ALU_79	9	7	7	12
ALU_86	6	6	10	10
ALU_88	9	5	7	10
ALU_92	10	4	8	14
ALU_02	12	9	12	22
ALU_11	9	6	10	13
ALU_13	8	6	11	13
ALU_30	20	14	20	26
ALU_96	7	6	8	11

Table S2: Elevational position of the cline centres (in meters a.s.l., when applicable), width and the log-likelihood of the fitted clines based on the average q-values.

CLINE	Blumenstein	Salvan	Filfalle
**Centre**	**1569**	**1404**	**1186**
Centre confidence interval	1490-1631	1379-1429	1179-1209
**Width**	**320**	**185**	**13**
Width confidence interval	142-591	137-252	0.16-124
**Ln L**	**-10.84**	**-4.73**	**-1.8**

Supplementary Figures

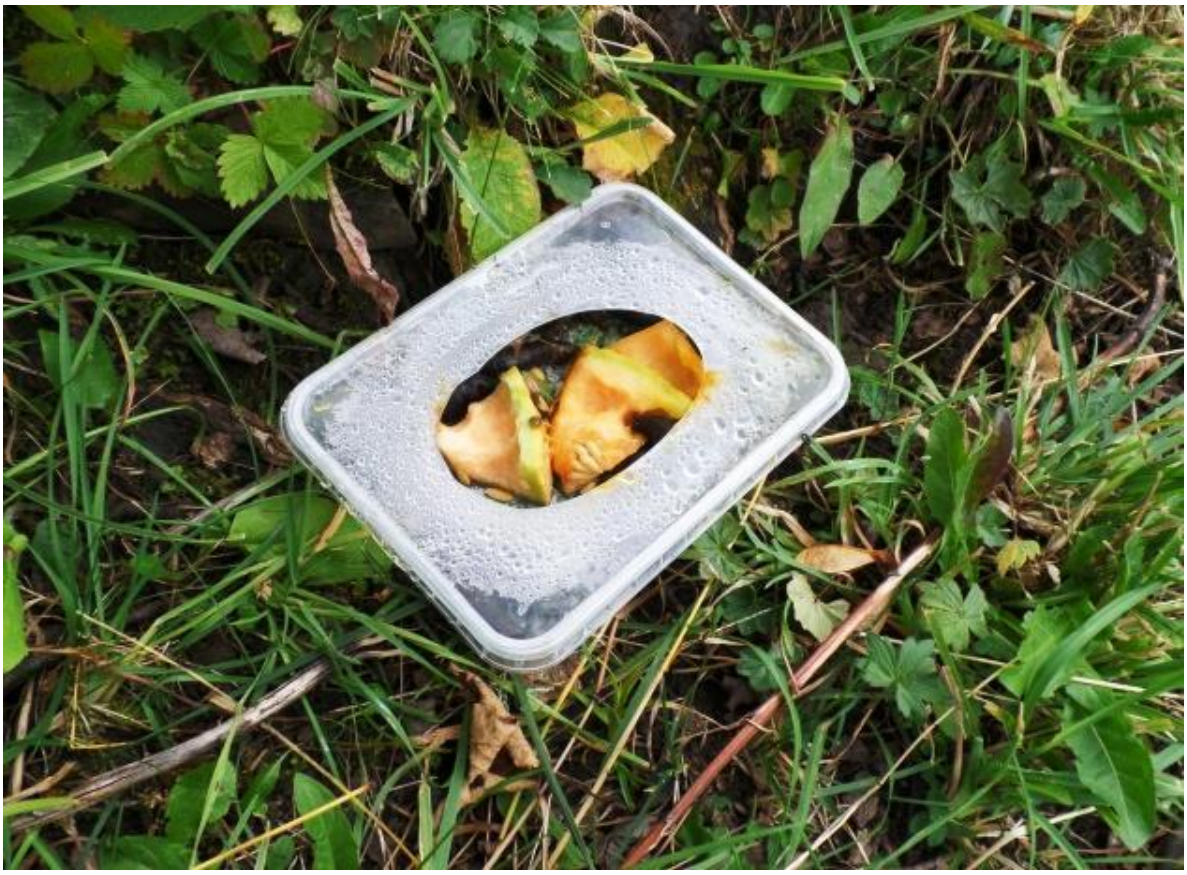

Figure S1: Example of a self-developed slug trap with *Arion* slugs inside attracted by a piece of fruit. The trap was created from a plastic box with an opening in the lid that is on the lower part covered with an anti-slug paste (IRKA, Germany) to allow slugs crawl into the box but prevent their escape.

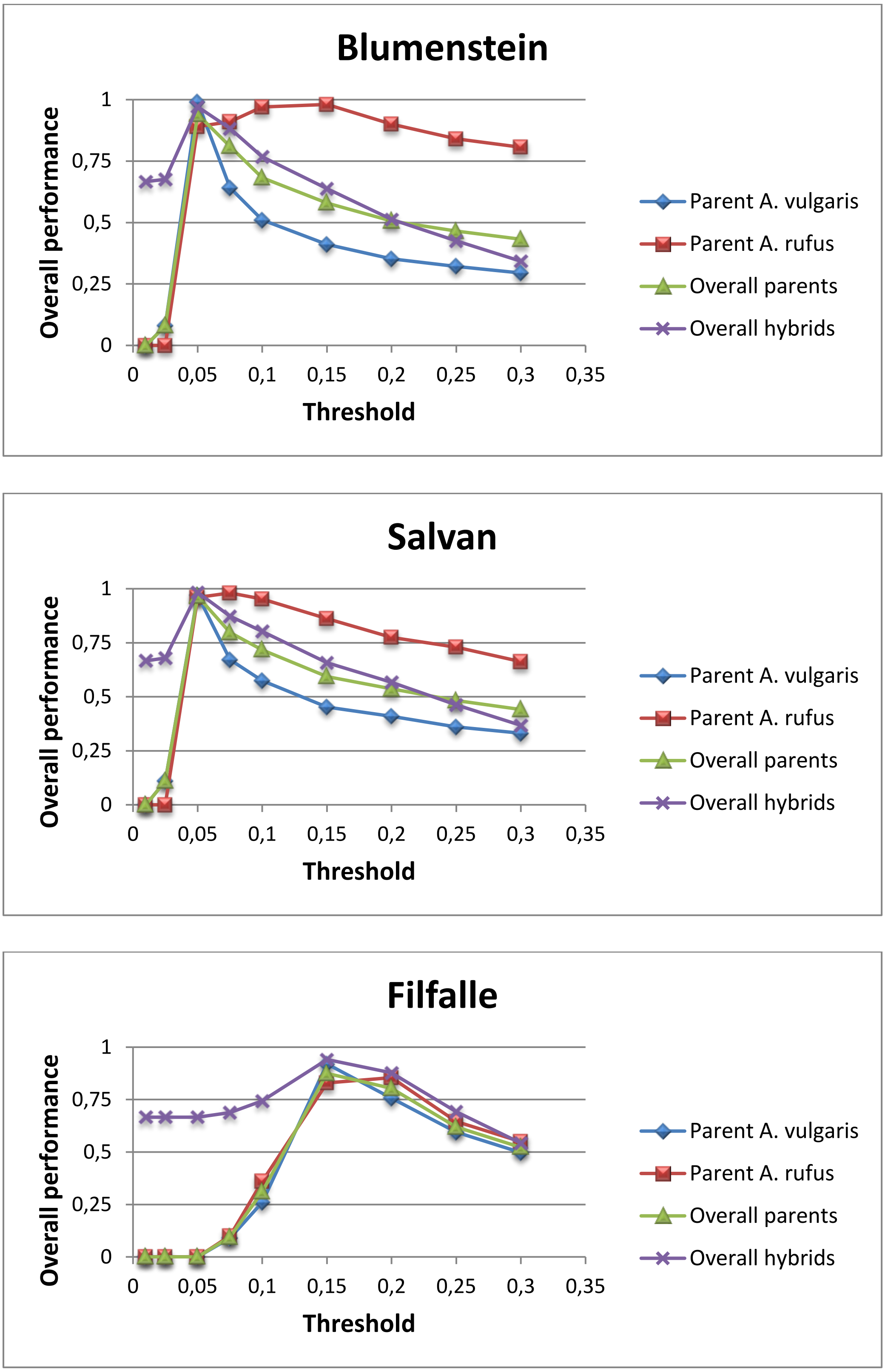

Figure S2: The efficiency and accuracy (overall performance) of detecting parental and hybrid (F1, backcrosses) individuals simulated by HYBRIDLAB for different STRUCTURE threshold q-values. The best result is achieved when the threshold is set to 0.05 for Blumenstein (top) and Salvan (middle), and 0.15 for Filfalle (bottom).

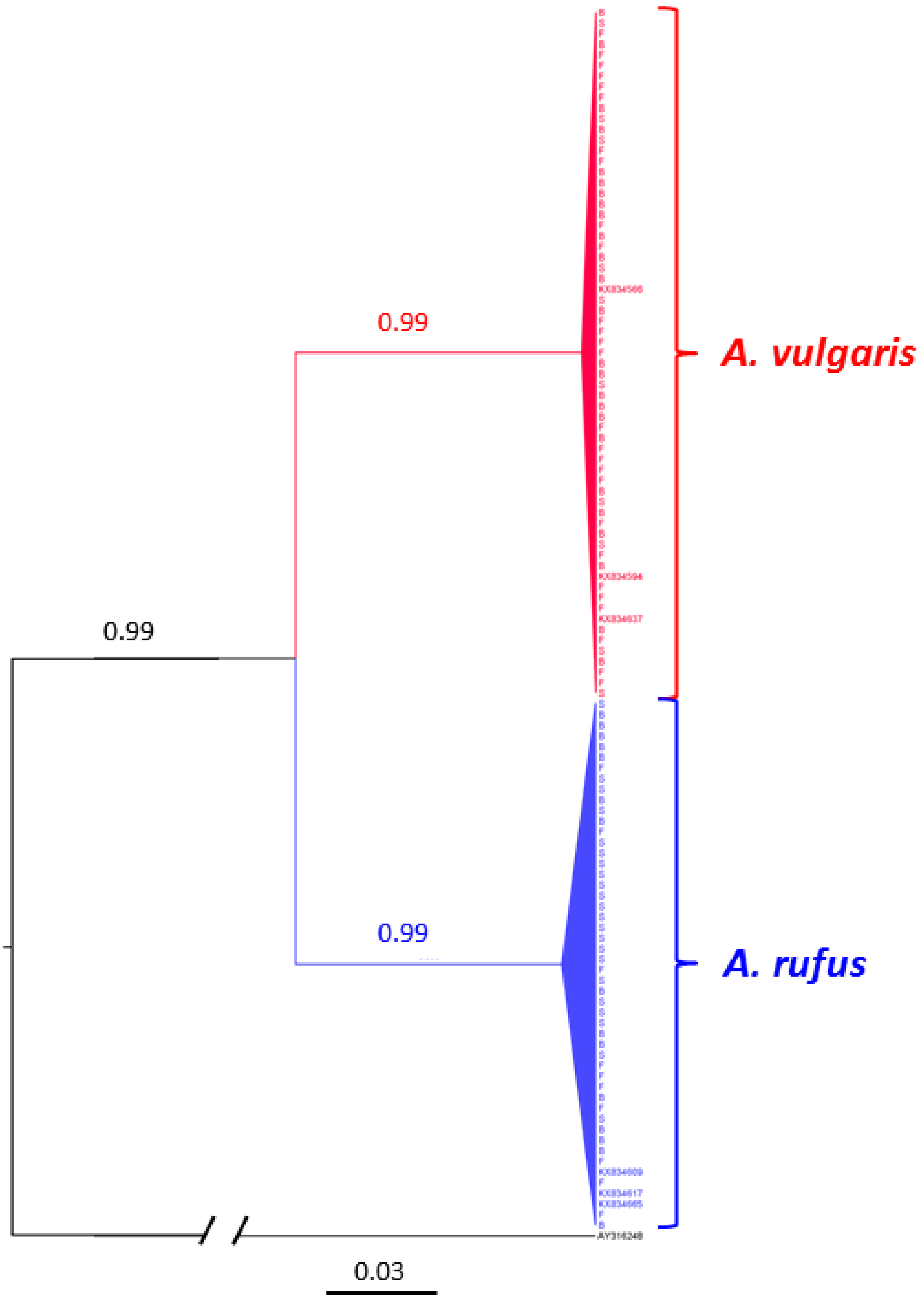

Figure S3: Bayesian reconstruction of phylogenetic relationships based on ND1 sequences of *Arion* sp. slugs from the three altitudinal transects (B – Blumenstein, F – Filfalle, S – Salvan; Tables 1-3) and three reference sequences from our previous study (Zemanova *et al.* 2016) representing each species, with *A. subfuscus* as outgroup. Sequences in the monophyletic cluster identified as *A. vulgaris* (60 individuals) are highlighted in red and *A. rufus* (45 individuals) in blue. The tree was collapsed for clarity. Posterior probabilities are displayed above the branches and the scale bar indicates the number of nucleotide substitutions per site.

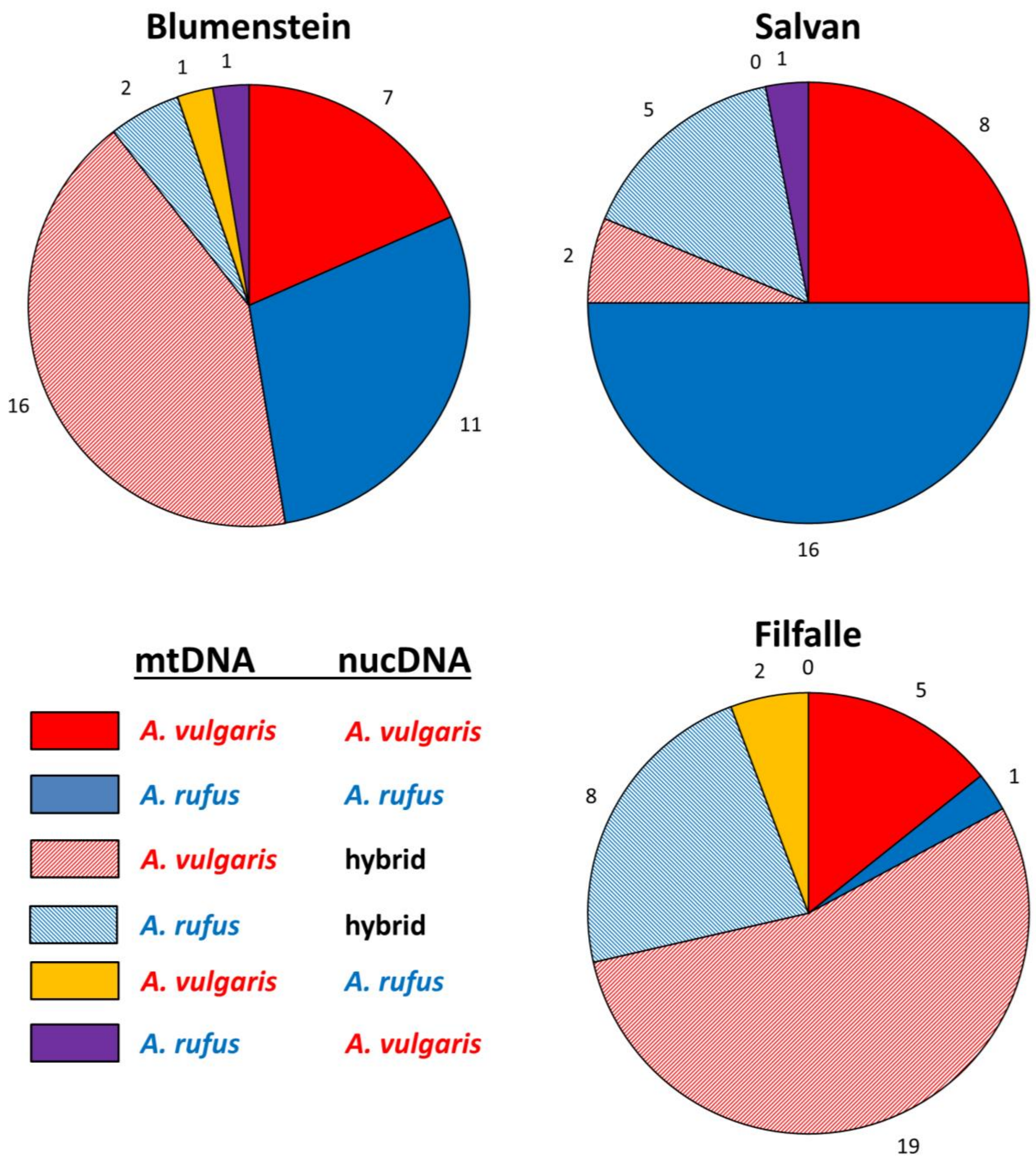

Figure S4: Species assignment for 105 slugs analysed for mitochondrial DNA (mtDNA) and nuclear DNA (nucDNA), represented for each transect separately. Hybrids were assigned based on the thresholds identified in HYBRIDLAB. The number of slugs assigned to each category is indicated.

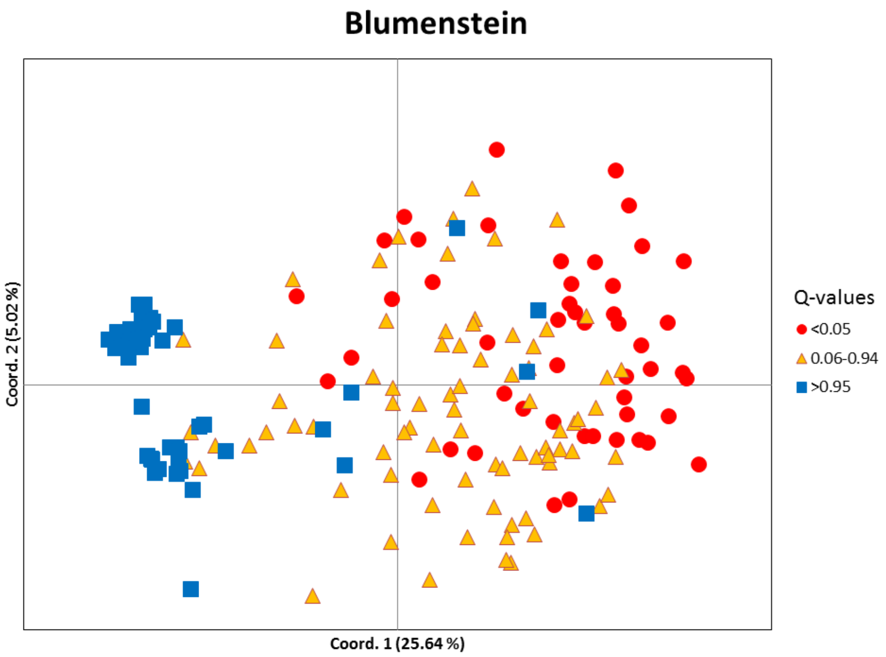

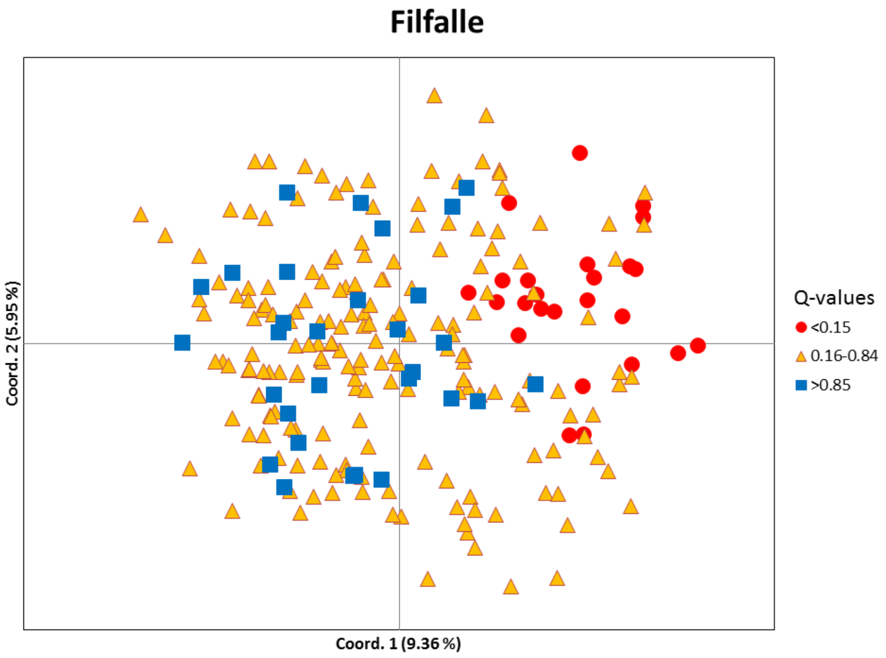

Figure S5: Principal coordinate analyses for *Arion* sp. in the Blumenstein (top) and Filfalle (bottom) transects based on microsatellite genotypes. Individuals are colour-coded according to the q-value thresholds identified in HYBRIDLAB: red – pure *A. vulgaris*, blue – pure *A. rufus*, yellow – admixed individuals. The percentage of the total variation in the dataset that is explained by each principal coordinate is given in parentheses. See Figure 3 for the Salvan transect.

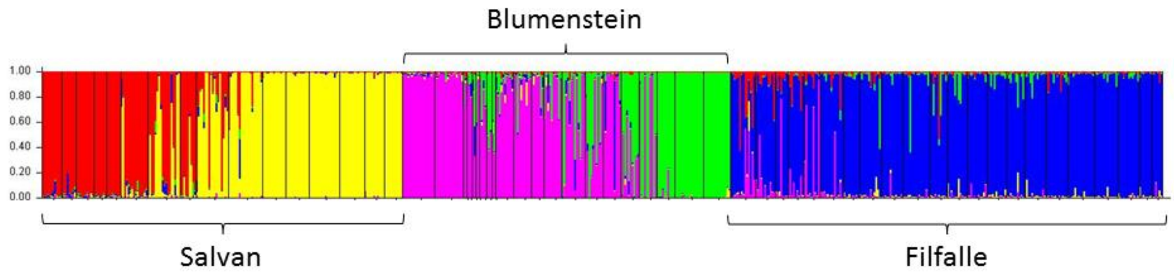

Figure S6: STRUCTURE plot for slugs from all three transects analysed together, with the most likely number of clusters (determined by delta K) K=5. Each individual is represented by a single vertical line, with sampling locations separated by a black line. The three transects were clearly separated and also *A. vulgaris* was distinguished from *A. rufus* in the Salvan and Blumenstein transects.
